# A Redox-Sensitive Cysteine Is Required for PIN1At Function

**DOI:** 10.3389/fpls.2021.735423

**Published:** 2021-12-16

**Authors:** Benjamin Selles, Tiphaine Dhalleine, Alexis Boutilliat, Nicolas Rouhier, Jérémy Couturier

**Affiliations:** ^1^Université de Lorraine, INRAE, IAM, Nancy, France; ^2^Institut Universitaire de France, Paris, France

**Keywords:** cysteine oxidation, parvulin, peptidyl-prolyl cis/trans isomerases, plant, thioredoxin

## Abstract

Parvulins are ubiquitous peptidyl-prolyl isomerases (PPIases) required for protein folding and regulation. Among parvulin members, Arabidopsis PIN1At, human PIN1, and yeast ESS1 share a conserved cysteine residue but differ by the presence of an N-terminal WW domain, absent in PIN1At. In this study, we have explored whether the cysteine residue of Arabidopsis PIN1At is involved in catalysis and subject to oxidative modifications. From the functional complementation of yeast *ess1* mutant, we concluded that the cysteine at position 69 is mandatory for PIN1At function *in vivo*, unless being replaced by an Asp which is found in a few parvulin members. This result correlates with a decrease of the *in vitro* PPIase activity of non-functional PIN1At cysteinic variants. A decrease of PIN1At activity was observed upon H_2_O_2_ treatment. The *in vitro* oxidation of cysteine 69, which has an acidic p*K_a_* value of 4.9, leads to the formation of covalent dimers that are reduced by thioredoxins, or to sulfinic or sulfonic acid forms at higher H_2_O_2_ excess. These investigations highlight the importance of the sole cysteine residue of PIN1At for activity. The reversible formation of an intermolecular disulfide bond might constitute a protective or regulatory mechanism under oxidizing conditions.

## Introduction

**Figure 1:**
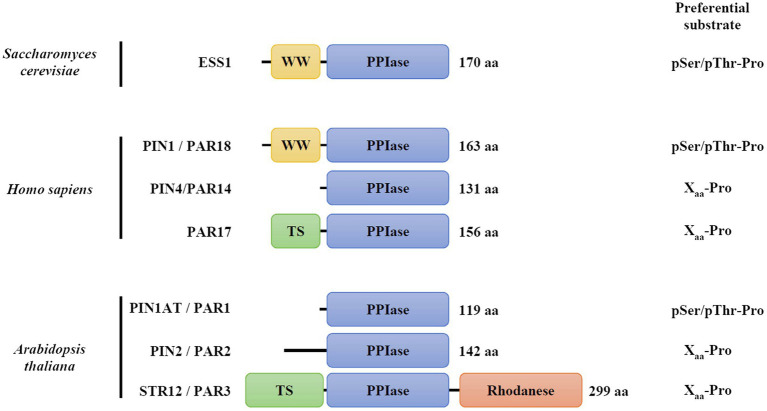
Modular organization and substrate specificity of parvulin family members. The corresponding accession numbers are: *Saccharomyces cerevisiae* ESS1, P22696; *Homo sapiens* PIN1/PAR18, Q13526; *H. sapiens* PIN4/PAR14, Q9Y237; *H. sapiens* PIN4/PAR17, Q9Y237; and *Arabidopsis thaliana* PIN1At/PAR1, Q9SL42; *A. thaliana* PIN2/PAR2, Q9FE18; *A. thaliana* PAR3/STR12, Q93WI0. TS refers to as targeting sequence to mitochondria in PAR17, or to chloroplasts in STR12/PAR3. Information about substrate preferences were retrieved from Fanghänel and Fischer (2004).

The *cis-trans* isomerization of the peptide bond between a prolyl residue and the preceding amino acid (Xaa-Pro) is considered as a molecular switch involved notably in the regulation of protein function (Lu et al., 2007). Peptidyl-prolyl isomerase (PPIase) proteins catalyze this reaction and the PPIase superfamily is composed of four non-homologous protein families named cyclophilins, FK506-binding proteins (FKBPs), PP2A phosphatase activators (PTPA), and parvulins (Fanghänel and Fischer, 2004; Jordens et al., 2006; Lu et al., 2007). Parvulins are widespread proteins that were initially identified in *Escherichia coli* as PAR10 (or PpiC) protein (Rahfeld et al., 1994). In *Saccharomyces cerevisiae*, ESS1 represents the sole parvulin ortholog (Arevalo-Rodriguez et al., 2004), while two orthologs, PIN1/PAR18 and PIN4/PAR14, are present in metazoans (Uchida et al., 1999; Thapar, 2015). In human, an alternative initiation transcription site for the PIN4 gene leads to the synthesis of a second isoform referred to as PAR17 protein, which is targeted to mitochondria due to the presence of an N-terminal matrix targeting sequence (Figure 1; Mueller et al., 2006; Kessler et al., 2007). In photosynthetic organisms, three genes encode proteins with a parvulin domain (PAR1/PIN1At, PAR2/PIN2 and PAR3/STR12; Kouri et al., 2009; He, 2012; Selles et al., 2019; Moseler et al., 2020).

The parvulin family is divided into two categories depending on the presence of a phosphorylated amino acid (either pSer or pThr) preceding the prolyl residue in their substrates (Yaffe et al., 1997; Yao et al., 2001; Fanghänel and Fischer, 2004). Yeast ESS1, human PIN1 and Arabidopsis PIN1At orthologs display the highest affinity for phosphorylated substrates (Yao et al., 2001; Fanghänel and Fischer, 2004; Behrsin et al., 2007). Human PIN1 and yeast ESS1 are formed by two domains, the parvulin domain being fused at the C-terminus of a small domain (approximately 30 amino acids) containing two Trp residues and thus referred to as WW domain (Lu et al., 1996). While the isolated parvulin domain is active *in vitro* (Zhou et al., 2000), the structural analysis of human PIN1 suggests a potential function of the WW domain in protein folding and stability or in the binding of phosphorylated substrates (Ranganathan et al., 1997). Despite recent advances, two molecular mechanisms have been proposed for the *cis-trans* proline isomerization reaction catalyzed by parvulins. In the so-called covalent mechanism, the nucleophilic properties of a presumed catalytic cysteine (Cys113 residue in human PIN1) would be required. It is notably supported by crystallographic data of PIN1 protein in complex with a synthetic peptide showing that four residues (Cys113, His59, His157, and Ser154, human PIN1 numbering, Supplementary Figure S1) are in interaction with the substrate, the Cys113 residue being in a thiolate form (Ranganathan et al., 1997). In the alternative non-covalent mechanism, it is proposed that the negative charge, rather than the nucleophilic property of Cys113 residue, is involved in the destabilization of the partial double bond character of the peptide bond (Behrsin et al., 2007). In fact, a few parvulin members do not have this cysteine but an Asp instead (Supplementary Figure S1). A variant of human PIN1 in which Cys113 is substituted by an aspartic acid did fully complement a yeast *ess1* strain unlike variants in which it is substituted by a serine or an asparagine (Behrsin et al., 2007). These data rather support the non-covalent catalytic mechanism but raise the question about the exact role of this conserved cysteine residue. Actually, the Cys113 residue of human PIN1 is sensitive to peroxide-dependent oxidation, the formation of sulfenic, sulfinic, or sulfonic acids abolishing protein activity *in vitro* (Chen et al., 2015; Innes et al., 2015). This may suggest also a possible redox regulation of the catalytic mechanism of human PIN1 and other parvulin orthologs containing this cysteine.

In plants, the essential PIN1At from *Arabidopsis thaliana* (Arabidopsis) represents the best studied isoform (Landrieu et al., 2000, 2002; Xi et al., 2016). PIN1At is formed only by the parvulin domain in which a single cysteine residue (Cys69) is present and corresponds to Cys113 of human PIN1 (Figure 1, Supplementary Figure S1). Biochemical approaches have highlighted the capability of PIN1At to perform *cis-trans* prolyl isomerization *in vitro* (Landrieu et al., 2000; Yao et al., 2001). By catalyzing this reaction on specific protein targets, the AGL24 and SOC1 master regulators, or the PIN1 auxin efflux carrier, PIN1At proved to be important for flowering time transition and gravitropism (Torti and Fornara, 2012; Xi et al., 2016). While the role of Cys69 residue for PIN1At function has not been yet investigated, several redox proteomic studies have revealed that it is likely subject to redox post-translational modifications, such as sulfenylation and persulfidation (Liu et al., 2014; Aroca et al., 2017; Wei et al., 2020).

In this study, we have performed a biochemical and functional analysis of PIN1At focusing on the role of the Cys69 residue. The functional complementation of the yeast *ess1^H164R^* mutant using cysteinic variants indicates that the Cys69 residue is mandatory for PIN1At function *in vivo*. However, the possible substitution by an aspartate demonstrates that this is rather the charge than the nucleophilic property of the cysteine that is required for the catalytic mechanism. Using fluorescent alkylating reagent, the determination of an acidic p*K_a_* value for Cys69 is consistent with a deprotonated state in the cell. Consequently, it is sensitive to an H_2_O_2_-mediated oxidation which primarily formed a disulfide-bridged homodimer that is reduced *in vitro* by the thioredoxin (TRX) system. Hence, the reversible formation of an intermolecular disulfide bond may be seen as a protective or regulatory mechanism of PIN1At activity.

## Materials and Methods

### Heterologous Expression in *E. Coli* and Purification of Recombinant Proteins

The sequence coding for Arabidopsis PIN1At (At2g18040) was cloned between the *Nde*I and *Bam*HI restriction sites of pET12a and pET15b, enabling the expression of untagged and N-terminal His-tagged recombinant proteins, respectively. Cys69 residue was substituted into serine, aspartate, or asparagine using the pET15b-PIN1At plasmid as a template, mutagenic oligonucleotides, and the QuikChange site-directed mutagenesis kit (Agilent Technologies). The corresponding variants were named PIN1At C69S, C69D, and C69N. All primers used are listed in Supplementary Table S1.

Protein expression was performed in the *E. coli* BL21 (DE3) strain, containing the pSBET plasmid, which allows expression of the tRNA recognizing AGG and AGA codons. Cell cultures were progressively amplified up to 2.4L in LB medium supplemented with 50μg/mL of required antibiotics (ampicillin and kanamycin) and grown at 37°C. Protein expression was induced at exponential phase by adding 100μM isopropyl β-D-thiogalactopyranoside for 4h at 37°C. After centrifugation (20min at 6,380×*g*), the cell pellets were resuspended in about 20mL of 30mM Tris-HCl pH 8.0, 1mM EDTA, and 200mM NaCl (TE NaCl buffer) for the untagged PIN1At protein or 50mM Tris-HCl pH 8.0, 300mM NaCl, and 10mM imidazole for His-tagged versions of PIN1At and conserved at −20°C.

Cell lysis was performed by sonication (3×1min with intervals of 1min), and the soluble and insoluble fractions were separated by centrifugation for 30min at 27,216×*g*. The purification of untagged PIN1At protein was carried out in three steps. The soluble fraction was precipitated by ammonium sulfate in two steps (from 0 to 40% and then to 80% of the saturation) separated by centrifugation for 30min at 27,216×*g*. The 40–80% ammonium sulfate-precipitated fraction was subjected to gel filtration chromatography (ACA44 gel, Biosepra) equilibrated with TE NaCl buffer. After dialysis of the fractions of interest against TE buffer and concentration, the sample was applied to a DEAE-Sephacel ion exchange column equilibrated with TE buffer. The recombinant PIN1At protein passed through the DEAE column and was concentrated and dialyzed by ultrafiltration (Amicon cells, YM10 membrane) under nitrogen pressure and stored in TE buffer at −20°C. Since untagged PIN1At cysteinic variants were insoluble, they were expressed as 6-Histidine-tagged proteins. For His-tagged proteins, the soluble fraction was directly loaded on Ni^2+^ affinity columns (Sigma-Aldrich). After extensive washing, the recombinant proteins were eluted by adding 50mM Tris-HCl pH 8.0, 300mM NaCl, and 250mM imidazole. The recombinant proteins were concentrated and dialyzed as above and stored in TE buffer at −20°C. Protein concentrations were determined spectrophotometrically using a molecular extinction coefficient at 280nm of 6,990M^−1^ cm^−1^. Other recombinant proteins used in this work such as Arabidopsis NTRB and poplar TRXh1 and h3 have been purified as described previously (Jacquot et al., 1994; Behm and Jacquot, 2000).

### Yeast Complementation

The sequences coding for PIN1At and its C69S, C69D, and C69N variants were cloned between the *Bam*HI and *Xho*I restriction sites of a p426-TDH3 plasmid using primers listed in Supplementary Table S1 (Mühlenhoff et al., 2010). The *S. cerevisiae* BY4741 (MATa; ura3Δ0; leu2Δ0; his3Δ1; met15Δ0) and *ess1^H164R^* (MATa; ura3Δ0; leu2Δ0; his3Δ1; met15Δ0; ess1-H164R:kanMX) strains were obtained from Euroscarf. Empty or recombinant p426-TDH3 plasmids were introduced in yeast cells by the LiAc method (Gietz and Schiestl, 2007). Cells were grown on synthetic dropout medium with 2% glucose but lacking uracil as a selection marker. For functional complementation assay, tenfold serial dilutions of yeast cell cultures starting from an initial 0.05 O.D. at 600nm were spotted onto synthetic dropout solid medium lacking uracil. Plates were incubated at 20°C for 8days or 37°C for 4days. Complementation of the *ess1^H164R^* strain, which expresses a catalytically deficient enzyme that restricts growth at 37°C, was visualized by the restored ability of transformed cells to grow at this temperature.

### Peptidyl-Prolyl Isomerase Activity Measurements

The PPIase activity measurements were performed using the chymotrypsin-coupled method described previously with slight modifications (Behrsin et al., 2007). The synthetic substrate N-succinyl-Ala-Glu-Pro-Phe-p-nitroanilide (suc-AEPF-p-NA; Bachem) was dissolved at a stock concentration of 10mM in 450mM LiCl/trifluoroethanol. Reactions were performed at 5°C in a final volume of 500μL TE buffer with 10 to 500μM Suc-AEPF-p-NA in the presence or in the absence of 2μM of His-tagged PIN1At. After 1min of incubation, 25μg of chymotrypsin was added and the velocity of the reaction catalyzed by chymotrypsin onto *trans* conformers of the substrate was followed at 390nm. The concentration of released p-nitrolamine was determined using a molar extinction coefficient at 390nm of 13,300M^−1^ cm^−1^. Rates of PIN1At-catalyzed isomerization were obtained by subtracting the chemical isomerization measured prior to adding PIN1At from the combined chemical and enzyme-catalyzed rate obtained after addition of PIN1At. Apparent *k_cat_* and *K_m_* values were calculated by nonlinear regression using the Michaelis–Menten equation in GraphPad Prism.

To assess the impact of hydrogen peroxide treatment on PIN1At isomerase activity, 200μM PIN1At protein was reduced by 1mM DTT(red) for 1h in TE buffer. Reducing agent was removed by desalting (G25) in TE buffer. Control and oxidation mixtures were performed as follows: 20μM reduced proteins were incubated for 30min in the absence or in the presence of 500μM or 2mM H_2_O_2_ in TE buffer. The reversibility of treatment with 2mM H_2_O_2_ was tested by subsequently incubating treated protein with 10mM DTT(red) for 30min. Then, 2μM of untreated or H_2_O_2_-treated proteins were used, as described above, with 200μM Suc-AEPF-p-NA for isomerase activity. The results were expressed as a percentage of the activity of the untreated PIN1At protein.

### Determination of Cysteine p*K_a_*

Around 10mg of PIN1At was reduced using 30mM DTT(red) in 500μL of 30mM Tris-HCl pH 8.0 for 1h at 25°C. The reduced proteins were then desalted on a G25 column pre-equilibrated with 30mM Tris-HCl pH 8.0 buffer. The measurement of the p*K_a_* of Cys69 was taken at 25°C by incubating 10μM of reduced protein with 200μM 5-iodoacetamido fluorescein (fluorescein IAM) in 200μL of sodium citrate or phosphate buffer ranging from pH 2.0 to 7.5 as described previously (Zannini et al., 2017). Values were transformed into percentage of thiolate and fitted to the following nonlinear regression: % thiolate=Bottom+(Top−Bottom)/[1+10^(Log p*K_a_* – pH×HillSlope)].

### H_2_O_2_-Mediated Oxidation of PIN1At

In a final volume of 150μL of 30mM Tris-HCl pH 8.0 buffer, 150μM of reduced PIN1At was incubated or not in the presence of 500μM H_2_O_2_ for 30min at 25°C. After extensive dialysis against 10mM Tris-HCl pH 8.0, the untreated and H_2_O_2_-treated samples were analyzed by electrospray ionization mass spectrometry (ESI-MS) as described previously.

The sensitivity of PIN1At to H_2_O_2_ was evaluated by incubating reduced PIN1At (10μM) for 30min at 25°C in a final volume of 400μL of 30mM Tris-HCl pH 8.0 buffer in the presence of 0 to 1mM H_2_O_2_. Subsequent labeling of protein-free thiol groups by monobromobimane (mBBr) and fluorescence measurement were performed as described previously (Zannini et al., 2019).

### Size Exclusion Chromatography Coupled With Multi-Angle Light Scattering Analysis

300μL reaction mixtures containing 76μM (300μg of protein) reduced PIN1At proteins were incubated for 30min in 30mM Tris-HCl pH 8.0, 200mM NaCl without or with H_2_O_2_ at concentrations ranging from 100μM to 2.5mM. Reaction mixtures were then injected on Superdex200 10/300 column (GE Healthcare) connected to an ÄKTA-Purifier FPLC (GE Healthcare) and coupled to a MALS detector miniDAWN TREOS II (Wyatt) and a differential refractometer Optilab T-rEX (Wyatt). Data were integrated with ASTRA software to determine the oligomerization state and exact molecular masses.

### Determination of Midpoint Redox Potential (*E_m_*)

Oxidized PIN1At was prepared by incubating 1mM reduced protein with 16.7mM H_2_O_2_ for 30min in 300μL of HEPES 100mM pH 8.0 at 25°C before desalting on G25 column. The formation of disulfide-bridged PIN1At homodimer was then verified by ESI-MS analysis as described above. Oxidation–reduction titration by incubating 10μM of oxidized PIN1At for 2h in mixtures of oxidized and reduced DTT at 2mM was carried out as described previously (Selles et al., 2012).

### Cysteine Alkylation Shift Assay

Reduced PIN1At (10μM) was incubated at 25°C for 30min in the presence of 0 to 2.5mM H_2_O_2_ in 50μL of 30mM Tris-HCl pH 8.0 buffer. Protein-free thiol groups were alkylated with methoxy-PEG (mPEG)-maleimide of 2kDa as described previously before separating the protein mixture on non-reducing 15% SDS-PAGE (Zannini et al., 2017).

### TRX-Mediated Reduction of PIN1At Homodimer

Reduced PIN1At (16μM) was incubated at 25°C for 30min with 500μM H_2_O_2_ in 20μL of 30mM Tris-HCl pH 8.0 buffer. Then, the whole reconstituted TRX reducing system (200μM NADPH, 100nM Arabidopsis NTRB and 1μM poplar TRXh1 or h3) was added to the 20μL reaction and further incubated 30min at 25°C. The reaction was stopped by addition of non-reducing Laemmli buffer and protein mixtures were separated on non-reducing 15% SDS-PAGE.

## Results

### The Cysteine 69 Is Required for PIN1At Function and Activity Unless Replaced by an Aspartate

**Figure 2:**
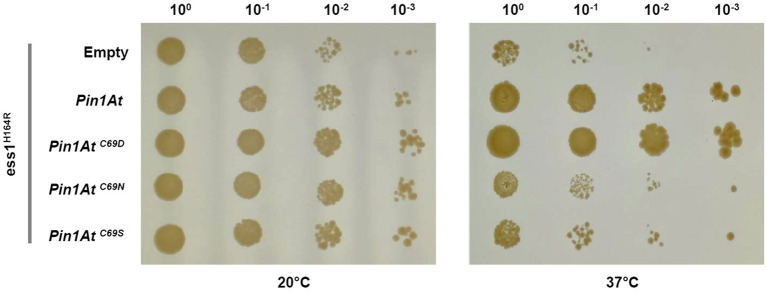
Functional complementation of *ess1^H164R^* strain by PIN1At and its variants. Cells expressing PIN1At and its C69D, C69N, and C69S variants (as assessed by RT-PCRs, Supplementary Figure S2) were plated as tenfold serial dilutions on SD - Ura medium. Functional complementation is visualized by the ability of transformed cells to grow at restrictive temperature (37°C) for 4days. A control assay shows cells grown for 8days at permissive temperature (20°C). The results shown here are representative of three independent experiments.

It was previously reported that plant parvulins were able to complement the yeast *ess1* strains even though they lack the N-terminal WW domain (Metzner et al., 2001; Yao et al., 2001). However, the importance of Cys69 for the physiological role of PIN1At was not examined. To this end, we tested the ability of three PIN1At variants to complement the temperature sensitive phenotype of *ess1^H164R^* strain (Wu et al., 2009). In these variants, the Cys69 was substituted to an aspartate, mimicking the residue present in a few isoforms, but also to serine and asparagine to compare with the work conducted on human PIN1 (Behrsin et al., 2007). Only PIN1At and its C69D variant restored the ability of *ess1^H164R^* strain to grow at the 37°C restrictive temperature (Figure 2).

To investigate further the role of Cys69 *in vitro*, the variants were expressed as 6-His-tagged recombinant proteins in *E. coli*, purified to homogeneity and the PPIase activity measured using Suc-AEPF-pNA peptide as a substrate since it was the preferred substrate of the *Lotus japonicus* PIN1 ortholog (Kouri et al., 2009). The steady-state catalytic parameters were determined for each recombinant protein using increasing substrate concentrations (0 to 500μM; Table 1; Supplementary Figure S3). All proteins exhibited PPIase activity with the Suc-AEPF-pNA peptide. While the turnover numbers were not much affected, the proteins differed in the apparent *K_m_* values for the substrate. This resulted in similar catalytic efficiencies for PIN1At and its C69D variant (1.0×10^4^ and 1.2×10^4^M^−1^ s^−1^, respectively) which are 7 to 18-fold higher than the ones of the C69N and C69S variants (1.4×10^3^ and 5.8×10^2^M^−1^ s^−1^, respectively; Table 1). From these results, we can conclude that an aspartic acid can fulfill the function of the PIN1At cysteine residue at least on the synthetic substrate used here but also in a cellular context as exemplified by the yeast complementation.

**Table 1:** Kinetic parameters of PPIase activity of PIN1At and its C69S, C69N, and C69S variants.

	PIN1At	PIN1At C69D	PIN1At C69N	PIN1At C69S
*k*_cat_ (s^−1^)	0.75±0.08	1.12±0.20	0.40±0.10	0.56±0.07
*K_m_* (μM)	74±29	90±9	271±113	961±335
*k*_cat_/*K_m_* (M^−1^ s^−1^)	1.0±0.3×10^4^	1.2±0.4×10^4^	1.4±1×10^3^	5.8±1.2×10^2^

Steady-state kinetic parameters were calculated from activity measurements shown in Supplementary Figure S3 by fitting them to nonlinear regression using the Michaelis–Menten equation.

### H_2_O_2_ Inhibits PIN1At Activity and Promotes the Formation of Disulfide-Bridged Homodimer

**Figure 3:**
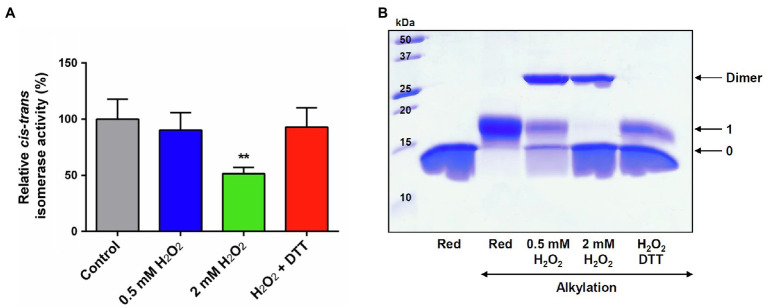
H_2_O_2_-dependent inactivation of PPIase activity of PIN1At. **(A)** The PPIase activity of reduced PIN1At treated or not with 25- or 100-fold excess H_2_O_2_ was measured using chymotrypsin-coupled assay in the presence of 200μM of suc-AEPF-p-NA as substrate. The activity of the untreated PIN1At was set to 100%. The data represent the mean±SD and *t*-test *p* value (^**^p<0.05) of six independent experiments. **(B)** Reduced PIN1At protein (20μM) was treated or not or not with 25- or 100-fold excess H_2_O_2_ for 30min. Protein sample treated with 100-fold excess H_2_O_2_ was incubated with 10mM DTT(red) for 30min after protein precipitation and alkylation of free thiol groups with 2kDa mPEG-maleimide (except in one reference line), protein mixtures were separated on non-reducing SDS-PAGE 15%. The numbers on the right correspond to the number of thiols that remained reduced upon treatment and thus were alkylated with mPEG maleimide.

We sought to assess next the effect of an H_2_O_2_ treatment on the PPIase activity of PIN1At. The activity was measured after treating a reduced protein with a 25- or 100-fold excess H_2_O_2_. A two-fold decrease was observed with the largest excess (Figure 3A). The subsequent treatment of H_2_O_2_-treated protein with DTT restored the full PPIase activity of PIN1At suggesting that a reversible redox modification was responsible for the inhibition of protein activity. The redox state of protein used for activity assays was analyzed by non-reducing SDS-PAGE after alkylation of the remaining free thiol groups with 2kDa mPEG-maleimide that enables to discriminate reduced and oxidized forms of protein (Figure 3B). At a 25-fold H_2_O_2_ excess, a covalent homodimer was preferentially observed as oxidized form with an oxidized monomeric form and a reduced monomeric form as well, while at a 100-fold H_2_O_2_ excess, PIN1At was fully oxidized with an equivalent amount of overoxidized monomeric and disulfide-bridged dimeric forms. A subsequent treatment with DTT reduced the dimeric form leading to the appearance of a reduced monomer in addition to the oxidized monomeric form that was not affected by DTT addition as expected (Figure 3B). These results reveal a relationship between inhibition of protein activity and its overoxidation and suggest that the disulfide-bridged dimer may be still active although less efficient than reduced PIN1At.

**Figure 4:**
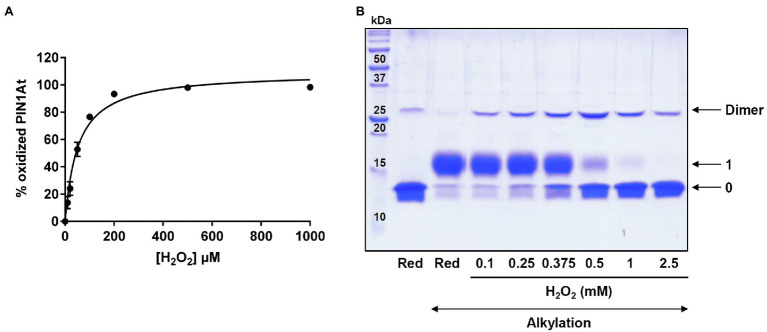
H_2_O_2_-dependent oxidation of PIN1At. **(A)** PIN1At is sensitive to oxidation by H_2_O_2_. The relative oxidation of 10μM PIN1At was evaluated in the presence of increasing concentrations of H_2_O_2_ for 30min. The remaining free thiols were alkylated with mBBr and quantified through measurement of the resulting fluorescence. The decrease of fluorescence was plotted against the oxidant concentration for determination of S_0.5_. The data represent the mean±SD of three separate experiments. **(B)** Reduced PIN1At protein (10μM) was treated or not with various concentrations of H_2_O_2_ for 30min. After protein precipitation and alkylation of free thiol groups with 2kDa mPEG-maleimide (except in one reference line), protein mixtures were separated on non-reducing SDS-PAGE 15%. The numbers on the right correspond to the number of thiols that remained reduced upon treatment and thus were alkylated with mPEG maleimide.

Further experiments were performed to specifically follow cysteine oxidation. Hence, 10μM pre-reduced PIN1At was incubated with increasing H_2_O_2_ concentrations (from 0 to 1mM, i.e., up to a 100-fold excess) and then reacted with mBBr, a fluorescent molecule that alkylates thiol groups. We observed a progressive decrease of the fluorescence as the H_2_O_2_ concentration increases which reflects an increase of PIN1At oxidation (Figure 4A). From these results, we determined a S_0.5_ value of 52±5μM that corresponds to the H_2_O_2_ concentration necessary to reach half-maximal protein oxidation. The redox state of PIN1At after treatment with 10- to 250-fold H_2_O_2_ excess was further analyzed by non-reducing SDS-PAGE after alkylation with 2kDa mPEG-maleimide (Figure 4B). At the lowest H_2_O_2_ excess (10- to 20-fold corresponding to 0.1 and 0.25mM, respectively), a covalent homodimer was preferentially observed as oxidized form, while an oxidized monomeric form became predominant from a 50-fold excess (0.5mM). PIN1At was almost fully oxidized in the presence of 100-fold H_2_O_2_ excess and mostly under oxidized monomeric form. It does presumably correspond to sulfinic and/or sulfonic acid forms, as observed for human PIN1 (Chen et al., 2015).

To validate this assumption, we performed ESI-MS analysis of the reduced PIN1At (150μM) treated or not with a~3-fold H_2_O_2_ excess (500μM; Table 2; Supplementary Figures S4, S5). A single species was obtained for the reduced protein with a mass of 12,882.7Da. The decrease of approximately 131Da compared to the theoretical molecular mass corresponds clearly to the cleavage of the first methionine as expected from the presence of an alanine at the second position. For the H_2_O_2_-treated protein, we detected three monomeric forms (molecular masses of 12,882.8, 12,913.9Da, and 12,930.9Da) and a disulfide-bridged dimer (molecular mass of 25,765Da; Table 2; Supplementary Figure S5). Among monomeric forms, the increase of 31.2 and 48.2Da compared to the reduced monomer suggests the addition of two and three oxygen atoms, respectively, consistent with the formation of sulfinic and sulfonic acids. Overall, these results demonstrate that PIN1At forms disulfide-bridged dimers when H_2_O_2_ excess is low. This may prevent the irreversible oxidation of the sole cysteine residue present in PIN1At that is observed at higher H_2_O_2_ concentrations.

**Table 2:** Electrospray ionization mass spectrometry analysis of reduced and H_2_O_2_-treated PIN1At.

Theoretical mass	DTT treatment	H_2_O_2_ treatment	Delta masses
13,014.7Da	12,882.7Da	12,882.8Da	0.1Da
	–	12,913.9Da	31.2Da
	–	12,930.9Da	48.2Da
	–	25,765.4Da	

The corresponding ESI-MS spectra are shown as Supplementary Figures S4, S5.

**Figure 5:**
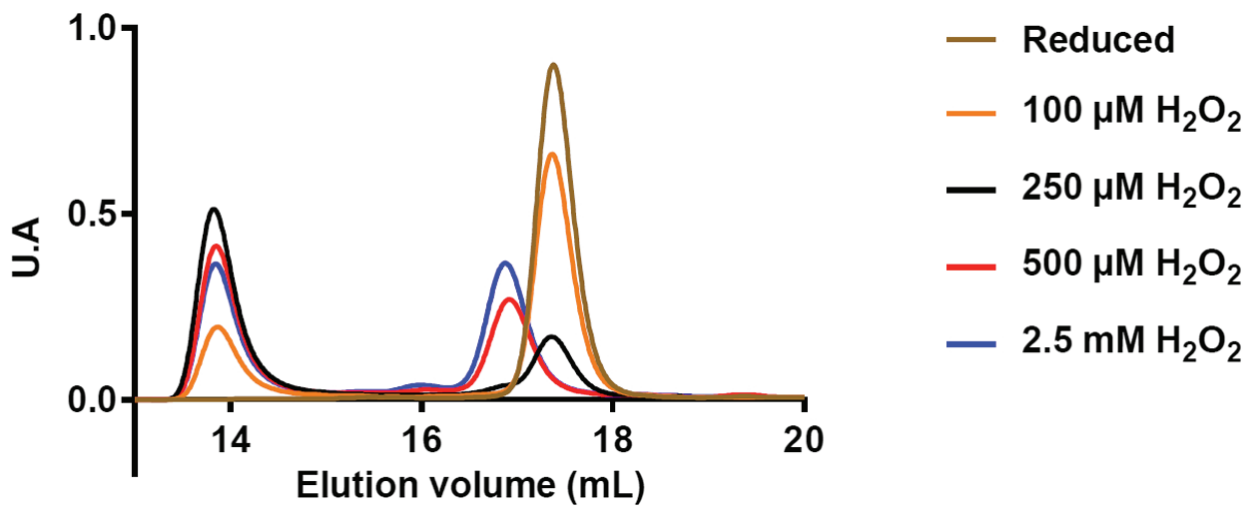
Protein redox state of PIN1At affects its oligomerization and hydrodynamic features. Analysis of reduced PIN1At treated or not with the indicated concentrations of H_2_O_2_ was performed by loading 300μg of protein on Superdex200 10/300 column coupled to a MALS detector and a differential refractometer.

These observations were completed by Size Exclusion Chromatography Coupled With Multi-Angle Light Scattering (SEC-MALS) analysis that enables to determine the oligomeric state and the molecular mass of proteins in solution. In these experiments, reduced PIN1At was treated with 1- to 30-fold excess H_2_O_2_ (0 to 2.5mM) and subsequently analyzed by SEC-MALS (Figure 5). Under reduced form, PIN1At eluted as a single peak with a calculated molecular mass of 12.3kDa (Figure 5). After H_2_O_2_ treatment, PIN1At eluted as two peaks with calculated molecular masses ranging from 12.2 to 12.5kDa and from 24.9 to 25.5kDa, thus corresponding to monomer and dimer forms, respectively. In the presence of 100μM H_2_O_2_ (1x excess), PIN1At remains mostly under a monomeric form whereas a dimeric form became predominant at 250μM H_2_O_2_ (3x excess). At higher H_2_O_2_ concentrations, the proportion of dimers did not increase further as already noticed in the cysteine alkylation experiments (Figure 4B). Moreover, the elution profile of the monomeric form is modified which suggested that cysteine overoxidation affected the globularity/hydrodynamic features of PIN1At (Figure 5). Hence, altogether these results indicate that a small excess of H_2_O_2_ promotes cysteine sulfenylation and subsequent formation of disulfide-bridged PIN1At dimer.

### Redox Properties of PIN1At and Reduction of the Covalent Dimer by Thioredoxin

**Figure 6:**
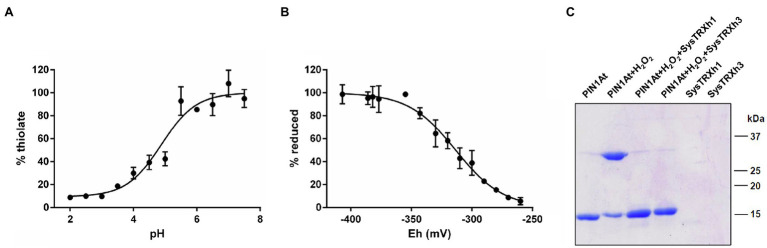
Redox properties of PIN1At. **(A)** The p*K_a_* values of sulfhydryl group Cys69 were determined by incubating 10μM of reduced PIN1At in buffers ranging from pH 2.0 to 7.5 in the presence of 200μM fluorescein-IAM which reacts with thiolates. The resulting fluorescence emission was expressed as % of maximal fluorescence as a function of the pH value of the solution. The obtained p*K_a_* value is the mean±SD of three separate experiments. **(B)** The titration of disulfide-bridged PIN1At dimer (10μM) was carried out using a total DTT concentration of 2mM for 2h at pH 7.0. Free thiol groups were labeled by mBBr and the resulting fluorescence emission was expressed as % of reduced protein and fitted to the redox potential of the solution. The obtained *E_m_* value is the mean±SD of three independent replicates. **(C)** Reduction of the H_2_O_2_ -mediated disulfide-bridged PIN1At dimer. Reduced PIN1At was treated with 500μM H_2_O_2_ and incubated or not for 30min in the presence of TRX system composed of 200μM NADPH, 100nM Arabidopsis NTRB, and 1μM poplar TRXh1 or h3. The reaction was stopped after 30min and protein mixtures separated on 15% SDS-PAGE in non-reducing conditions. The gel is representative of three experiments.

We sought to determine the p*K_a_* value of the conserved Cys69 of PIN1At using an alkylation method relying on fluorescent IAM (fluorescein IAM) that reacts with thiolate but not thiol groups. The pH-dependent reaction of reduced PIN1At with fluorescein IAM was followed in different buffers ranging from 2.0 to 7.5. From the titration curve, we obtained a p*K_a_* value of 4.9±0.1 (Figure 6A) indicating that Cys69 of PIN1At will be predominantly present as thiolate at physiological pH and thus susceptible to redox modifications pending it is exposed.

Next, we aimed at determining the redox midpoint potential (*E_m_*) of the intermolecular disulfide bridge formed upon PIN1At dimerization. We thus performed titration with mixtures of reduced and oxidized DTT and determined an *E_m_* value of −313±3mV at pH 7.0 (Figure 6B). This negative value is in the range of those determined for TRX partners and this prompted us to evaluate the ability of a reconstituted physiological TRX system comprising NADPH, Arabidopsis NTRB, and poplar TRXh1/h3 to catalyze the *in vitro* reduction of the disulfide-bridged PIN1At dimer. Adding catalytic amounts of each TRX isoform successfully reduced PIN1At dimers (Figure 6C). This suggests the existence of a protective mechanism by which the formation of this disulfide prevents the overoxidation of the sole cysteine residue of PIN1At under adverse physiological conditions where H_2_O_2_ is formed. Reduction by the TRX system might then constitute the second part of this protective mechanism for a rapid enzyme reactivation.

## Discussion

In the present study, we have coupled functional complementation with biochemical approaches to decipher the role of the conserved cysteine residue in cysteine-containing parvulins from plants using PIN1At from *A. thaliana* as a model.

### Cys69 Is Required for the Physiological Function of the Monodomain Parvulin PIN1At

The major difference between multidomain (human PIN1 and yeast ESS1) and monodomain (plant isoforms) parvulins is the presence of the so-called WW domain (Landrieu et al., 2000; Yao et al., 2001). While the WW domain of human PIN1 is important for the complementation of the *ess1^H164R^* strain, the observed complementation by monodomain parvulins from plants lacking the WW domain suggests that plant parvulin proteins adopt a different substrate recognition mechanism (Landrieu et al., 2000; Zhou et al., 2000; Yao et al., 2001). It was proposed that a four-amino-acid insertion within the PPIase domain of monodomain parvulins might substitute for the need of the WW domain as in multidomain parvulins (Yao et al., 2001). In human PIN1, it was reported that the domain interactions increase protein affinity toward peptide ligands (Matena et al., 2013) and that substrate recognition *via* the WW domain induces dynamic changes that promote PPIase domain activity (Campitelli et al., 2018; Lee and Liou, 2018).

Another question relates to the presence of a cysteine or an aspartate in the active site of parvulins and the role played by these residues. We have thus investigated the role of Cys69 of PIN1At by performing yeast functional complementation with PIN1At variants (C69D, C69S, and C69N) mimicking those of human PIN1 (C113D, C113S, and C113N variants) used in a previous study (Ranganathan et al., 1997; Behrsin et al., 2007). Heterologous expression of PIN1At C69S and C69N did not rescue the growth defects of the yeast strain. It seems that the lower PPIase activity of both C69N and C69S variants is not sufficient to support yeast growth (Figure 2; Table 2) as already observed with the human PIN1 C113N and C113S variants, the latter displaying a 20- to 50-fold decrease of PPIase activity *in vitro* (Ranganathan et al., 1997; Behrsin et al., 2007).

Both PIN1At C69D and human PIN1 C113D variants are functional and restore the growth of the *ess1^H164R^* mutant in restrictive conditions (Figure 2; Behrsin et al., 2007). Nevertheless, replacing Cys69 by an aspartate residue did not affect the substrate affinity and activity of PIN1At while the human PIN1 C113D variant displays a 6-fold decreased isomerase activity but apparently remains functional enough (Gemmill et al., 2005; Behrsin et al., 2007). This raises the question about the conservation of the catalytic mechanism and/or structural differences between monodomain and multidomain parvulins. Structural analysis of human PIN1 C113S and C113A variants have demonstrated that the dynamics in hydrogen bond network in the active site involving two conserved histidine residues and a threonine residue (Mueller et al., 2011) is modified by the nature of residue present at the position where the cysteine is (Barman and Hamelberg, 2014; Ikura et al., 2019). The effect of C113D mutation in human PIN1 has also been attributed to the putative disruption of the hydrogen bond between His59 and His157 which most probably affects the binding of the phosphate moiety of the substrates (Barman and Hamelberg, 2014; Xu et al., 2014; Wang et al., 2015; Chang et al., 2016). Since these two His are also present in PIN1At, it may be that the presence of the WW domain which helps in the positioning of the substrate in multidomain parvulins is at the origin of the different effects caused by cysteine substitution (Landrieu et al., 2002; Barman and Hamelberg, 2014; Xu et al., 2014). In any case, the fact that a Cys to Asp permutation is allowed at this position supports a mechanism where the Cys is rather required for maintaining the hydrogen-bond network than for playing a direct role in catalysis.

### PIN1At Cysteine Residue Is Prone to Oxidation

In plants, several members of the PPIase superfamily such as cyclophilin 20–3 (Laxa et al., 2007), FKBP20-2 (Lima et al., 2006) or FKBP13 (Gopalan et al., 2004; see Vasudevan et al., 2015 for review) are subject to redox post-translational modifications (PTMs). In previous studies, PIN1At has also been identified as sulfenylated in response to H_2_O_2_ treatments (Liu et al., 2014; Wei et al., 2020) and persulfidated in standard growing conditions (Aroca et al., 2017). Similarly, the Cys113 of human PIN1 is prone to redox PTMs such as persulfidation and irreversible oxidation (sulfinylation and sulfonylation), in addition to other PTMs such as phosphorylation, acetylation, and SUMOylation (Chen et al., 2015; Innes et al., 2015; Longen et al., 2016; Chen et al., 2020). Of interest, the irreversible oxidation of human PIN1 was associated with various pathologies as mild cognitive impairment or Alzheimer’s disease (Butterfield et al., 2006; Sultana et al., 2006) and induces a decrease of reactivity and mislocalization impairing the physiological function of the protein (Chen et al., 2015).

Unlike human PIN1, H_2_O_2_ did not inhibit completely the PPIase activity of PIN1At using Suc-AEPF-pNA peptide as substrate (Figure 3A; Chen et al., 2015). *In vitro* investigation revealed that oxidation of human PIN1 induced protein instability and aggregation (Bayer et al., 2003). While the protein stability was not investigated here, SEC-MALS analysis did not identify an oxidation-dependent aggregation of PIN1At after an H_2_O_2_ treatment. Furthermore, the previous study of PIN1At 3D structure by NMR revealed that Cys69 undergoes very little changes upon addition of the small phosphorylated Cdc25 peptide AcEQPLpTPVTDL (Landrieu et al., 2002). According to the activity assays, it appears that an overoxidized monomer is inactive, whereas the disulfide-bridged dimer is still active *in vitro* and does not abolish the interaction with the small Suc-AEPF-pNA peptide (Figure 3). Nevertheless, we consider possible that it might prevent protein–protein interactions with larger molecules such as Arabidopsis auxin transporter PIN1, and MADS-domain transcription factors, AGL24 and SOC1 (Wang et al., 2010; Xi et al., 2016) and affect its physiological function.

The results obtained with both PIN1At and human PIN1 suggest that the sulfenylation formed in response to H_2_O_2_ may be a general behavior of cysteine-containing parvulins. In the case of PIN1At, this H_2_O_2_ sensitivity correlates with the low p*K_a_* value of 4.9 of Cys69 as it was proposed for human PIN1 (Behrsin et al., 2007) but not experimentally confirmed. Given the rather instable and reactive nature of sulfenic acids, this is only an intermediate toward the formation of a disulfide-bridged dimer in PIN1At. Whether this is a unique feature among parvulins remains to be investigated further because it was not described for human PIN1 which thus becomes overoxidized. Overoxidation of PIN1At occurs also *in vitro* when the H_2_O_2_ excess increases. Hence, disulfide bond formation could represent a mechanism to protect the sole thiol group of PIN1At against irreversible oxidation by H_2_O_2_. Alternatively, the cysteine of human PIN1 and PIN1At may be protected by the formation of persulfides which would occur if for instance hydrogen sulfide reacts with the sulfenic acid (Cuevasanta et al., 2015). The fact that protein persulfidation protects against cysteine oxidation has been already illustrated for several proteins (Wedmann et al., 2016; Moseler et al., 2021). For a fully protective and reversible mechanism, reduction of these oxidized species should occur. The *in vitro* data presented here suggest that TRX may be the physiological reductant of PIN1At dimer, the redox potential of the intermolecular disulfide being in the range of those usually reported for TRX targets.

## Conclusion

The data indicate that cysteine-containing parvulins are prone to oxidation which would not be the case of aspartate-containing parvulins which are based on the mutational analyses performed on PIN1At and human PIN1 globally equally well active. The formation of covalent dimers in PIN1At might represent a regulatory mechanism of protein activity and a protective mechanism against overoxidation that may be used by some other cysteine-containing parvulins. If experimentally confirmed, persulfidation would be a good protective alternative for proteins that cannot dimerize.

## Data Availability Statement

The raw data supporting the conclusions of this article will be made available by the authors, without undue reservation.

## Author Contributions

BS and JC contributed to conceptualization. BS, TD, and AB contributed to investigation. BS, NR, and JC contributed to validation, formal analysis, and writing-review and editing. NR and JC contributed to supervision. JC contributed to project administration and funding acquisition. All authors have read and agreed to the published version of the manuscript.

## Funding

This work and the salary of BS and AB were supported by the French National Research Agency (ANR) as part of the “Investissements d’Avenir” program (ANR-11-LABX-0002-01, Lab of Excellence ARBRE) and by grant no. ANR-16-CE20-0012.

## Conflict of Interest

The authors declare that the research was conducted in the absence of any commercial or financial relationships that could be construed as a potential conflict of interest.

## Publisher’s Note

All claims expressed in this article are solely those of the authors and do not necessarily represent those of their affiliated organizations, or those of the publisher, the editors and the reviewers. Any product that may be evaluated in this article, or claim that may be made by its manufacturer, is not guaranteed or endorsed by the publisher.
