# Supplementary material for: A Redox-Sensitive Cysteine Is Required for PIN1At Function
Source: Front Plant Sci. 2021 Dec 16;12:735423. doi: 10.3389/fpls.2021.735423 (PMC8716364; doi:10.3389/fpls.2021.735423)
Supplement: Supplementary file 1 [file Data_Sheet_1.PDF]

# *Supplementary Material*

# A redox-sensitive cysteine is required for PIN1At function

**Benjamin Selles [1,3], Tiphaine Dhalleine [1], Alexis Boutilliat [1], Nicolas Rouhier [1] and Jérémy Couturier [1,2*]**

[1]Université de Lorraine, INRAE, IAM, F-54000 Nancy, France

[2]Institut Universitaire de France, France

[3]present address: Université de Lorraine, CNRS, IMoPA, F-54000 Nancy, France

*** Correspondence:**
Jérémy Couturier
jeremy.couturier@univ-lorraine.fr

**TABLE S1. Primers used for cloning, site-directed mutagenesis and RT-PCR experiments.** The *Nde*I, *Bam*HI and *Xho*I restriction sites used for cloning are underlined in the primers. The mutagenic codons are in bold.

| **Name** | **Sequence** |
|---|---|
| PIN1At for *Nde*I | 5’ CCCCCCCCCATATGGCGTCGAGAGACCAA 3’ |
| PIN1At rev *Bam*HI | 5’ CCCCGGATCCTCAAGCTGTTCTCTTAATGAT 3’ |
| PIN1At for *Bam*HI | 5’ CCCCCGGATCCATGGCGTCGAGAGACCAA 3’ |
| PIN1At rev *Xho*I | 5’ CCCCCCCTCGAGTCAAGCTGTTCTCTT 3’ |
| PIN1At D27D for | 5’ AAAGCGTCGTGGAAG**GAC**CCAGAAGGGAAGATT 3’ |
| PIN1AT D27D rev | 5’ AATCTTCCCTTCTGG**GTC**CTTCCACGACGCTTT 3’ |
| PIN1At C69D for | 5’ ACTCGTGTTTCTGAC**GAT**AGCTCTGCTAAACGC 3’ |
| PIN1AT C69D rev | 5’ GCGTTTAGCAGAGCT**ATC**GTCAGAAACACGAGT 3’ |
| PIN1At C69N for | 5’ ACTCGTGTTTCTGAC**AAT**AGCTCTGCTAAACGC 3’ |
| PIN1AT C69N rev | 5’ GCGTTTAGCAGAGCT**ATT**GTCAGAAACACGAGT 3’ |
| PIN1At C69S for | 5’ ACTCGTGTTTCTGAC**AGT**AGCTCTGCTAAACGC 3’ |
| PIN1AT C69S rev | **5’** GCGTTTAGCAGAGCT**ACT**GTCAGAAACACGAGT 3’ |
| ScGAS1 for *Spe*I | 5’ CCCCCACTAGTATGTTGTTTAAATCCCTT 3’ |
| ScGAS1 rev *Hind*III | 5’ CCCCCAAGCTTAACCAAAGCAAAACCGAC 3’ |

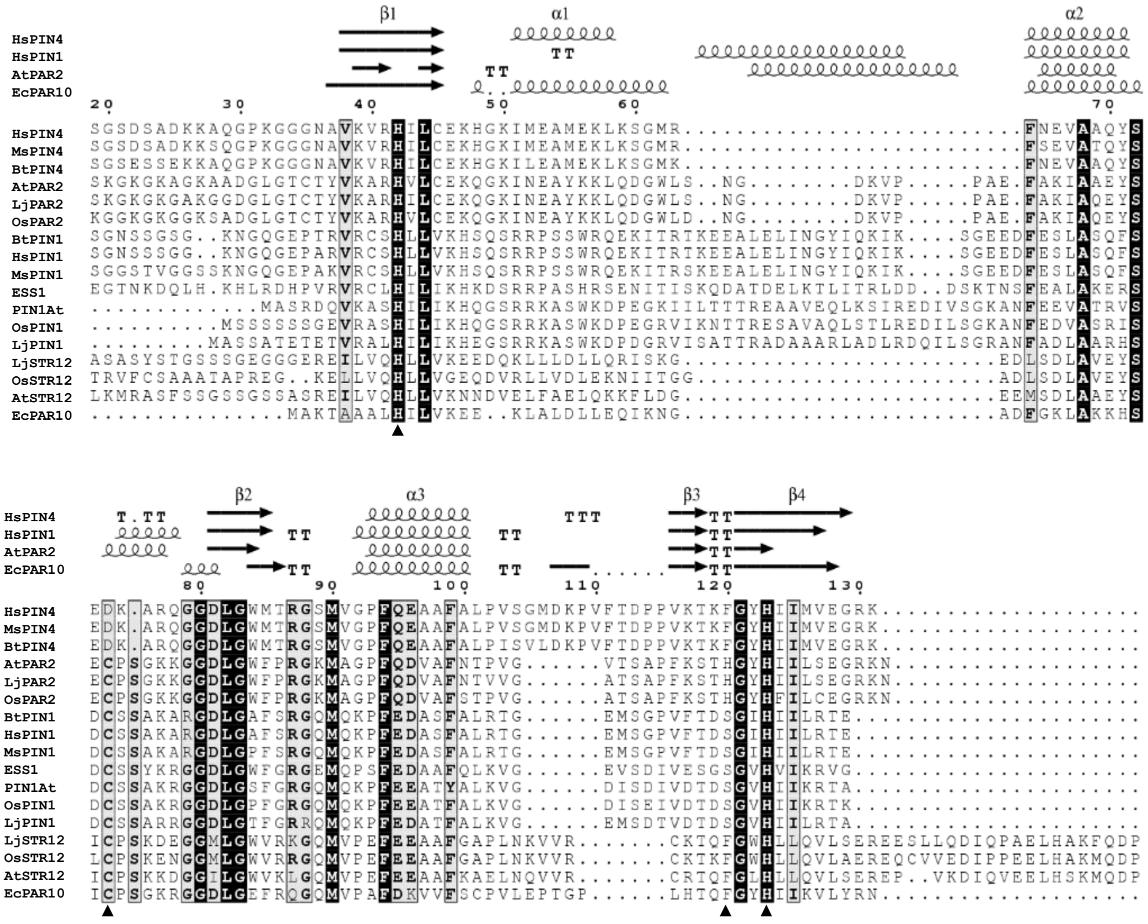


**FIGURE S1.** Multiple sequence alignment of parvulin orthologs from several model organisms (Hs, *Homo sapiens*; Bt, *Bos taurus*; Ms, *Mus musculus*; Ec, *Escherichia coli*; At, *Arabidopsis thaliana;* Lj, *Lotus japonicus*; Os, *Oryza sativa*). Arrows indicate amino acids that belong to the catalytic site. Sequences were retrieved from UniProt database (https://www.uniprot.org/) and aligned with CLUSTALW (https://npsa-prabi.ibcp.fr/cgi-bin/npsa_automat.pl?page=/NPSA/npsa_clustalw.html). The corresponding accession numbers are: yeast ESS1, P22696; HsPIN1, Q13526; HsPIN4, Q9Y237; PIN1At, Q9SL42; AtPAR2, Q9FE18; AtSTR12, Q93WI0; *Bt*PIN1, Q5BIN5; *Bt*PIN4, A6QPY8; *Ms*PIN1, Q9QUR7; *Ms*PIN4, Q9CWW6; EcPAR10, P0A9L5; LjPIN1, A7DX09; LjPAR2, A7DX10; LjSTR12, A7DX11; OsPIN1, Q7XTK0; OsPAR2, Q6ESK5 and OsSTR12, Q69WA8). Alignment output was generated by EsPrit3.0 (http://espript.ibcp.fr/). Secondary structure alignment was generated from PIN1At (PDB 1J6Y), EcPAR10 (PDB 1JNT), human PIN1 (PDB 4U84) and human PIN4 (PDB 3UI4).

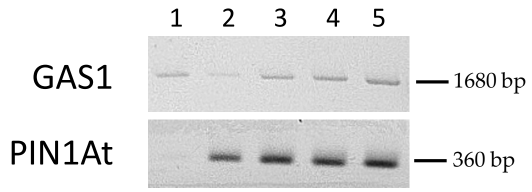


**FIGURE S2.** Transcript expression levels of PIN1At variants in yeast cells. Yeast *ess1*$^{H164R}$ cells were transformed with and empty *pRS426::pTDH3* (1), *pRS426::pTDH3::PIN1At* (2), *pRS426::pTDH3::PIN1AtC69D* (3), *pRS426::pTDH3::PIN1AtC69N* (4) or *pRS426::pTDH3::PIN1AtC69S* (5). Total RNA extraction was performed with the RNeasy Plant Mini kit (Qiagen) following manufacturer instructions. To obtain cDNA, 1 µg of total RNA was annealed with oligo-dT primers and reverse transcribed using Reverse Transcriptase (Biorad) followed by PCR amplification with a specific primer pair for PIN1At (PIN1At for *Nde*I and PIN1At rev *Bam*HI). Amplification of the yeast *GAS1/YMR307W* gene coding for a beta-1,3-glucanosyltransferase was used as control and amplified with a specific primer pair (ScGAS1 for *Spe*I and ScGAS1 rev *Hind*III). Primers used are listed in Table S1.

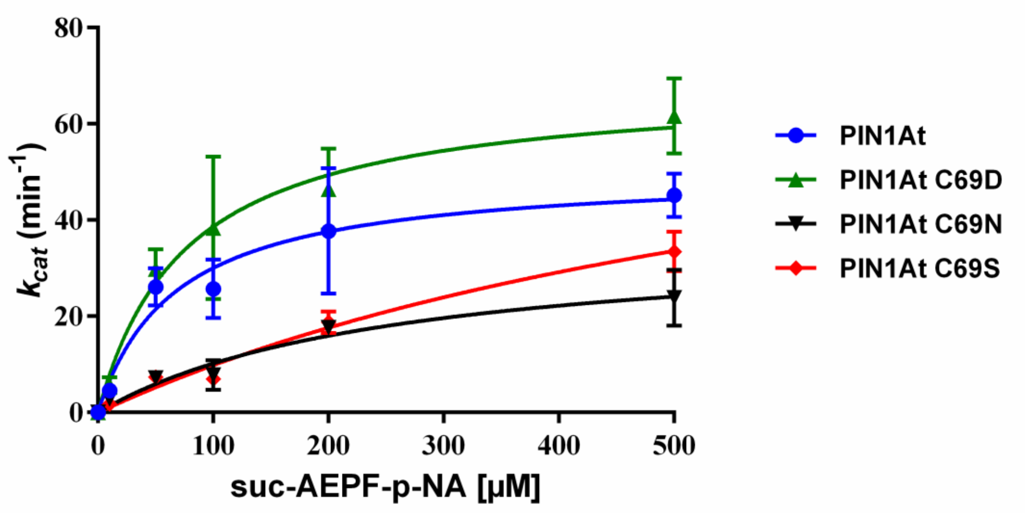


**FIGURE S3.** Determination of peptidyl-prolyl isomerase activity of PIN1At and its C69D, C69N and C69S variants. PPIase activity was assayed using a protease coupled method adapted from Behrsin et al., 2007. In brief, reactions were conducted at 5°C in 500µL 30 mM Tris HCl pH 8.0, 1 mM EDTA, with 25 µg α-chymotrypsin and 2 µM PIN1At proteins. After 15 s, various concentrations of suc-AEPF-p-nitroalinide peptide ranging from 10 to 500 µM were added to the mixture. The conformational-dependent activity of the α-chymotrypsin was then followed at 390 nm with ε = 13,300 $M^{-1}$ $cm^{-1}$. Rates of specific PIN1At-catalyzed isomerization were obtained by subtracting chemical isomerization measured in the absence of PIN1At. Kinetic parameters were calculated using a non-linear least-squares fit to the Michaelis–Menten equation in GraphPad Prism.

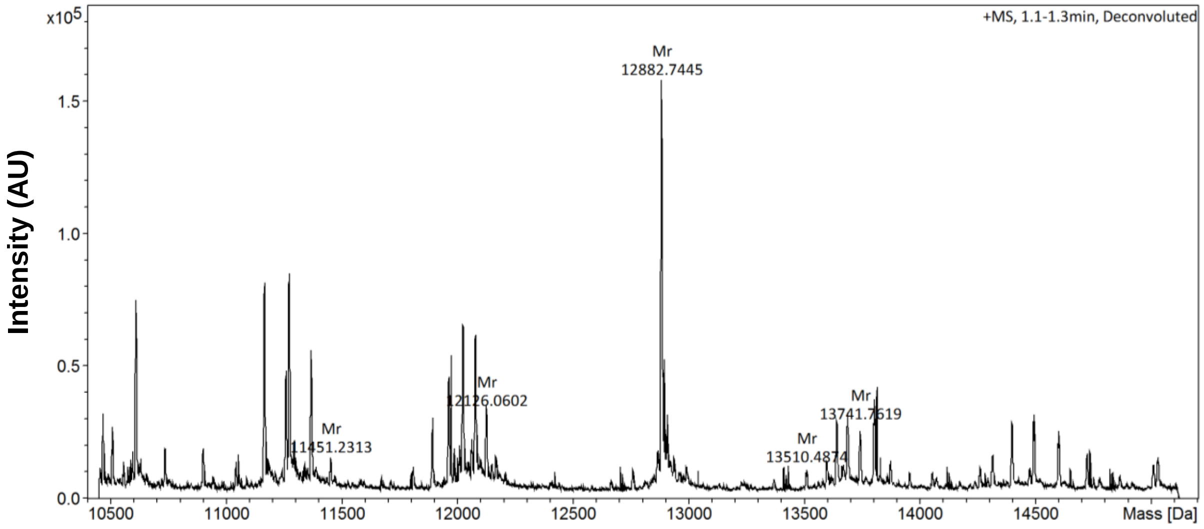


**FIGURE S4.** Electrospray ionization mass spectrometry analysis of reduced PIN1At. Deconvoluted mass spectrum of untagged PIN1At protein determined for a reduced protein as described in the "Materials and Methods" section. The species with a mass of 12882.7445 corresponds to the reduced PIN1At form.

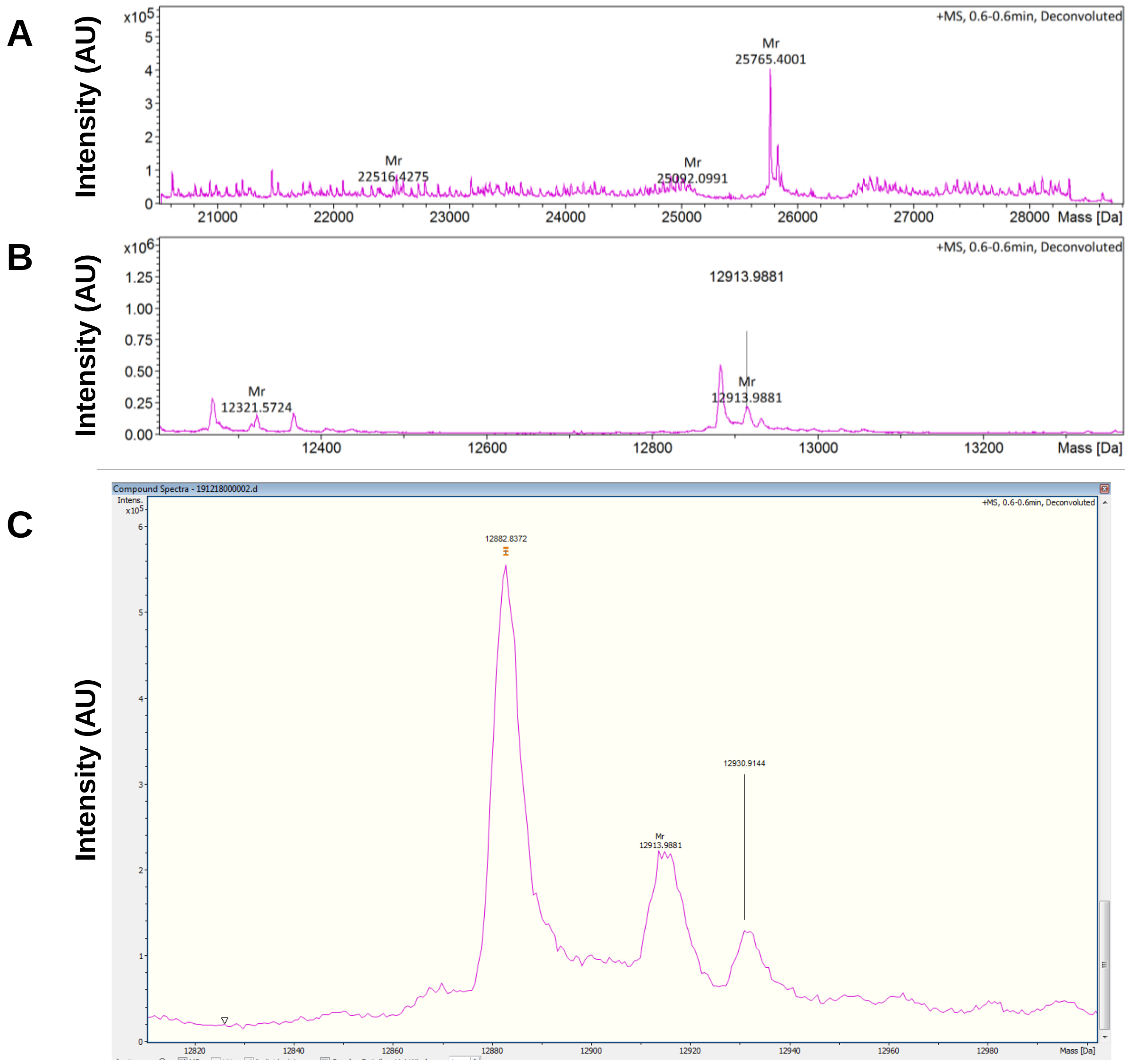


**FIGURE S5.** Electrospray ionization mass spectrometry analysis of oxidized PIN1At. Deconvoluted mass spectrum of untagged PIN1At protein determined for a reduced protein treated with 500 µM $H_2O_2$ for 1h as described in the "Materials and Methods" section. The species with a mass of 257652.4001 represents a disulfide-bridged dimer (A). Species with a mass of 12882.8372, 12913.9881 and 12930.9144 correspond to reduced, sulfinylated and sulfonylated forms, respectively (B and C).
